# Development of massive multilevel molecular dynamics simulation program, platypus (PLATform for dYnamic protein unified simulation), for the elucidation of protein functions

**DOI:** 10.1002/jcc.24318

**Published:** 2016-03-04

**Authors:** Yu Takano, Kazuto Nakata, Yasushige Yonezawa, Haruki Nakamura

**Affiliations:** ^1^ Research Center for State‐of‐the‐Art Functional Protein Analysis, Institute for Protein Research, Osaka University3‐2 Yamadaoka, SuitaOsaka565‐0871Japan; ^2^ Department of Frontier Sciences, Graduate School of Information SciencesHiroshima City University3‐4‐1 Ozuka‐Higashi, Asa‐Minami‐KuHiroshima731‐3194Japan; ^3^ 1st Government and Public Solutions DivisionNEC Corporation5‐7‐1, ShibaMinato‐KuTokyo108‐8001Japan; ^4^ High Pressure Protein Research Center, Institute of Advanced Technology, Kinki University930 Nishimitani KinokawaWakayama649‐6493Japan

**Keywords:** QM/MM‐MD simulation, massively parallel computations, K computer, speedup, parallelization ratio

## Abstract

A massively parallel program for quantum mechanical‐molecular mechanical (QM/MM) molecular dynamics simulation, called Platypus (PLATform for dYnamic Protein Unified Simulation), was developed to elucidate protein functions. The speedup and the parallelization ratio of Platypus in the QM and QM/MM calculations were assessed for a bacteriochlorophyll dimer in the photosynthetic reaction center (DIMER) on the K computer, a massively parallel computer achieving 10 PetaFLOPs with 705,024 cores. Platypus exhibited the increase in speedup up to 20,000 core processors at the HF/cc‐pVDZ and B3LYP/cc‐pVDZ, and up to 10,000 core processors by the CASCI(16,16)/6‐31G** calculations. We also performed excited QM/MM‐MD simulations on the chromophore of Sirius (SIRIUS) in water. Sirius is a pH‐insensitive and photo‐stable ultramarine fluorescent protein. Platypus accelerated on‐the‐fly excited‐state QM/MM‐MD simulations for SIRIUS in water, using over 4000 core processors. In addition, it also succeeded in 50‐ps (200,000‐step) on‐the‐fly excited‐state QM/MM‐MD simulations for the SIRIUS in water. © 2016 The Authors. Journal of Computational Chemistry Published by Wiley Periodicals, Inc.

## Introduction

Living things are enormously complex; however, they are composed of only a few different types of macromolecules, such as proteins, nucleic acids, polysaccharides, and lipids.^1^, ^2^ While all of the different kinds of biological macromolecules are essential for the functions of living things, proteins play diverse roles in biological processes to sustain life, as enzymes, antibodies, hormones, receptors, storage proteins, and structural proteins. A great deal of effort has been devoted toward understanding the mechanisms of these protein functions.

Recently, to elucidate the mechanisms in systems with complexities characteristic of biochemical processes, theoretical modeling has become an important tool, as a complement to experimental techniques. The computational investigation of the mechanisms of the functions accomplished by biomolecules is a rich and rapidly growing field in computational chemistry. In general, proteins function at the local region called the active site, where a catalytic chemical reaction occurs in an enzyme, an electron is received and released in an electron transport protein, or a chromophore is excited in a photoactive protein. Since chemical reactions and electron transfer are quantum‐mechanical phenomena, active sites should be treated in a quantum‐mechanical manner. In addition, the dynamical characteristics of proteins are also regarded as intrinsic fundamental properties of their functions, implying that the entire systems, including proteins and solvent molecules, should be considered in an investigation of the protein dynamics. Therefore, massive computer resources are required for both the quantum‐mechanical and dynamical analyses of proteins. Hybrid quantum mechanical–molecular mechanical (QM/MM) methods are applicable for the assessment of biological macromolecular systems, where local changes such as bond breaking and molecular excitation should be considered. These methods have become powerful tools for investigating biochemical reactions in proteins.^3^, ^4^, ^5^ In such simulations, the central reactive region is treated quantum mechanically, to allow key bonds to form and break, whereas the rest of the macromolecule as well as some explicit solvent molecules are modeled by MM methods, to make the calculations computationally feasible.

Nowadays, large‐scale calculations using massively parallel computing architectures are promising. Massively parallel computing utilizes thousands of computer nodes, each with many CPU or GPU cores. A supercomputer, the K computer, was manufactured at RIKEN in Japan.^6^, ^7^ The K computer system has 82,944 “SPARC64™ VIIIfx” CPUs designed and developed by Fujitsu.^8^ Each CPU is composed of 8 cores, a 6MB shared level 2 cache, and memory controllers. Peak performance of 128 GFLOPS (16 GFLOPS × 8 cores) is achieved at an operating frequency of 2 GHz with power consumption as low as 58W. An eight‐channel memory interface per CPU provides a peak memory bandwidth of 64 GB/s. In the K computer, the network that exchanges data such as computational results between CPUs also plays an important role. The K computer uses an network architecture called “Tofu (Torus Fusion)” for massive parallel computers.^9^ It ensures high data communication and fault tolerance. The network topology of the Tofu is a 6D mesh/torus. This enables the mutual interconnection of more than 80,000 CPUs. In June 2011, the K computer was ranked as the world's fastest supercomputer with 548,352 cores, and the full K computer system achieved 10 PetaFLOPs with 705,024 cores in November 2011.^10^ Most recently, the K computer is the fourth‐fastest at the TOP500 competition^11^ and the fastest at the Graph500 competition^12^ in November 2015.

In the present study, a hybrid MPI/OpenMP parallel code of QM/MM molecular dynamics (QM/MM‐MD) simulations was implemented within the Platypus program (PLATform for dYnamic Protein Unified Simulation) for massively parallel computations on the K computer in Japan, based on our previous QM/MM‐MD studies.^13^, ^14^ The performance of Platypus on the K computer was assessed for a bacteriochlorophyll dimer in the photosynthetic reaction center (DIMER). On‐the‐fly excited‐state QM/MM‐MD simulations were also performed for the chromophore of an ultramarine fluorescent protein, Sirius, (SIRIUS) in the aqueous state.

## Materials and Methods

### Features of the platypus program suites

**Figure 1:**
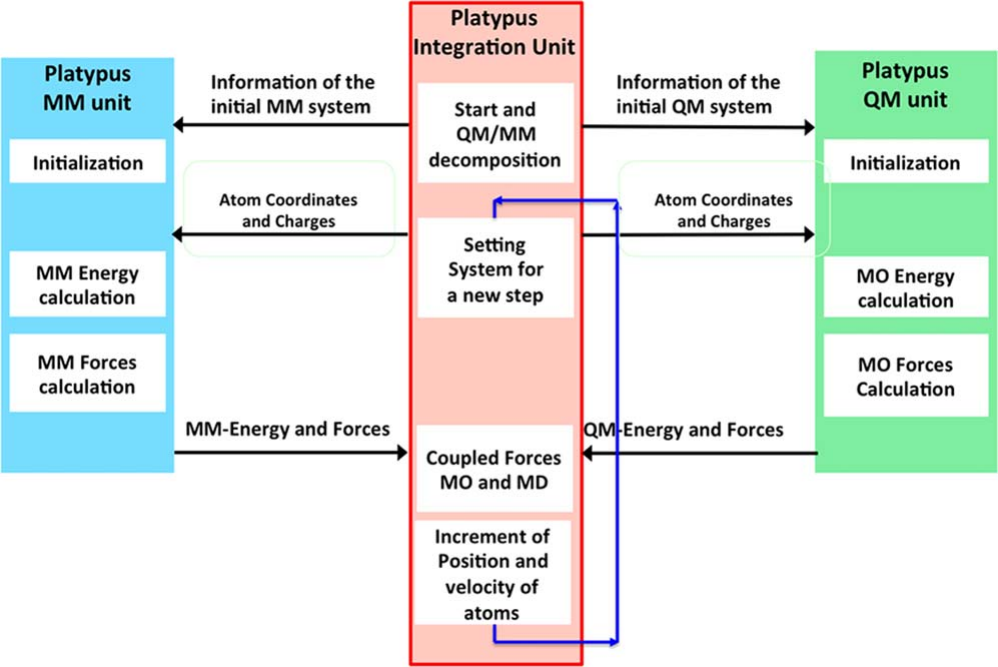
Data flow of Platypus in the QM/MM‐MD simulations.

The new program, Platypus, is composed of the Platypus‐QM unit, the Platypus‐MM unit, and the Platypus integration unit for the QM/MM calculation, as shown in Figure 1. The total energy of the whole system, *E*_tot_, is represented as

(1) $$E_{tot}=E_{QM}+E_{MM}+E_{QM/MM},$$

where the *E*_QM_ and *E*_MM_ terms denote the energies of the QM and MM parts, respectively. The *E*_QM/MM_ term includes the interaction energies between the QM and MM parts, and is composed of the electrostatic interaction, *E*_ele_, and the van der Waals interaction, *E*_vdW_, as

(2) $$E_{QM/MM}=E_{ele}+E_{vdW.}$$

The *E*_ele_ term is computed by the Platypus‐QM unit, and the *E*_vdW_ term is evaluated by the Platypus‐MM unit. In the Platypus‐QM unit, Hartree–Fock (HF), density functional theory (DFT), configuration interaction singles (CIS)^15^ and doubles (CIS(D)),^16^ second‐order Møller–Plesset perturbation theory (MP2),^17^ complete active‐space configuration interaction (CASCI), and complete active‐space self‐consistent field (CASSCF)^18^, ^19^ were implemented. In the post HF computations such as CIS, CIS(D), CASCI, and CASSCF, two electron integral transformation to molecular orbital is performed by the parallel‐driven algorithm, according to Ref. [ 19]. The AMBER ff96,^20^ ff99,^21^ and ff99SB^22^ force fields can be adopted in the Platypus‐MM unit.

For the QM‐MM boundary problem, the link atom method has been implemented in platypus. A hydrogen atom was put on the line that connected from QM boundary to MM boundary atoms. To avoid the additional unnatural degree of freedom in QM‐MM system, the length between the QM boundary atom and the hydrogen atom set to 1.10 Å. The hydrogen atom was then treated as a QM atom in the QM region. The charge redistribution method^23^ was also implemented in the platypus. The MM boundary atom and the atoms chemically bounded to the MM boundary atoms dose not electrostatically interact with the atoms in the QM regions. The omitted charge was redistributed to corresponding residue in the atoms of the MM regions. If at least one QM atom was involved in two, three, and four body bonded MM interactions from the MM system, the bounded MM interactions were applied to the QM atom.

The data flow of Platypus is shown in Figure 1. In the QM/MM calculation, Platypus first decomposes the system into the QM part and the MM part on the Platypus integration unit. The Platypus integration unit sends information including the atomic coordinates and charges to the Platypus‐QM and MM units. The Platypus‐QM and MM units compute the energies and forces and send them to the Platypus integration unit. On the integration unit, the computed QM and MM energies and forces are coupled, and the positions and velocities of the atoms are propagated. The above procedures are repeated.

The calculation of the QM part accounts for most of the total computational time required for the QM/MM‐MD simulations. Therefore, the Platypus‐QM unit was optimized by hybrid programming with a message passing interface (MPI) and open multi‐processing (OpenMP), and algorithms utilizing single instruction multiple data (SIMD) for massively parallel computing on a supercomputer such as the K computer, which has two 2‐way SIMD floating point multiply‐and‐add units per core. In addition, although the excited‐state QM calculations are required to use the post HF theories, such as CASSCF and CASCI,^18^, ^19^ the two electron integral transformation to molecular orbital sets is the most time consuming process for large‐scale calculations. The parallelization of the transformation was optimized for massively parallel computing on the K computer.^6^ We also implemented the eigen_sx routine of EigenExa^24^, ^25^ and the locally optimal block preconditioned conjugated gradient (LOBPCG)^26^ algorithm into the Platypus‐QM unit, to accelerate the computation of eigenvalue problems.

Although the performance of the Platypus was optimized by the MPI/OpenMP hybrid parallelization on the K computer, it can be also utilized on conventional PC clusters. The Platypus‐QM is available as free software from the website (http://www.islim.org/islim-dl_e.html). In the near future, the whole Platypus program suites will be available from the same website.

### Speedup and parallelization ratio

The parallelization performance of Platypus was measured by using speedup and parallelization ratio. Speedup (*S*) is the ratio of the elapsed times with a reference and *N* times as many core processors as the reference, and is evaluated as

(3) $$S=\frac{T_{1}}{T_{N}},$$

where the elapsed time measured with a reference is regarded as *T*_1_, and *T_N_* denotes the elapsed time of computation (*T*) with *N* times as many core processors as the reference.

The parallelization ratio (*P*)^27^, ^28^ means the parallelizable fraction of a program. If a program is not parallelized at all, then this value should be 0%. When a program runs *N* times as fast as measured with *N* times as many core processors as the reference, this value should be 100%. The ratio was computed from Amdahl's law,^29^ using eq. (4).

(4) $$P=\frac{N}{N-1}\left(1-\frac{T_{N}}{T_{1}}\right).$$

A speedup of more than *N* when using *N* times core processors is observed in parallel computing. It is called superlinear speedup. In the superlinear speedup, the parallelization ratio is evaluated to be more than 100%. Superlinear speedup often occurs due to the cache effect resulting from the different memory hierarchies of modern computers.

The Karp–Flatt metric,^30^ the experimentally determined serial fraction (*f*), can be derived with Amdahl's law^29^ as

(5) $$f=\frac{\frac{1}{S}-\frac{1}{N}}{1-\frac{1}{N}}.$$

The parallelization ratio can be represented using eq. (6).

(6) P= 1−f.

It means that the parallelization ratio corresponds to the Karp–Flatt metric in the present study.

### Targeted molecules

We conducted measurements of the speedup and the parallelization ratio of the energy and force calculations of DIMER by the Platypus‐QM unit. We tested the performance of the on‐the‐fly excited‐state QM/MM‐MD simulation by Platypus, using SIRIUS, the chromophore of a pH‐insensitive and photo‐stable ultramarine fluorescent protein, Sirius.^31^

**Figure 2:**
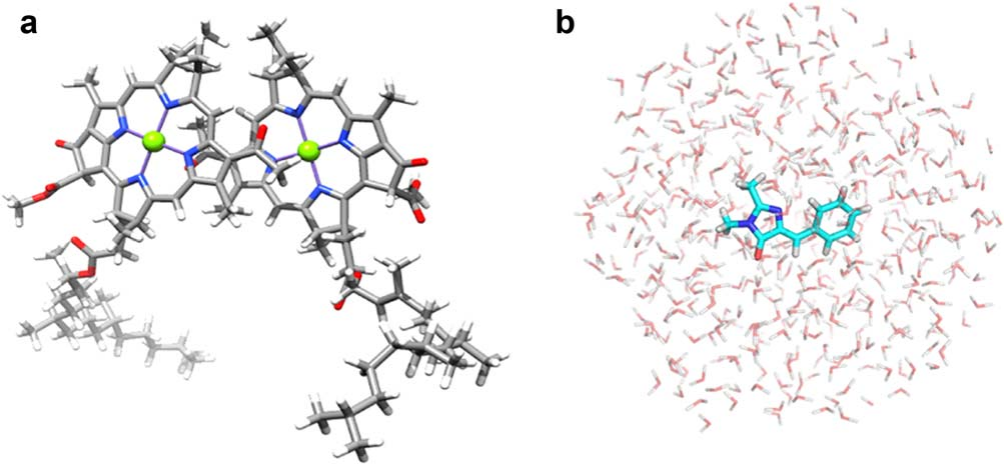
The DIMER model, consisting of 280 atoms and 30,904 point charges (**a**). The SIRIUS system, consisting of 1374 atoms, including SIRIUS (27 atoms) and 449 TIP3P waters (**b**).

The speedup and the parallelization ratio of the QM unit of Platypus were assessed for DIMER (280 atoms and 30,904 point charges). To include the electrostatic interaction of the surrounding protein in the electronic structure of the DIMER, the point charges of the surrounding protein subunits were taken from an AMBER ff96 force field.^20^ The atomic charges of the other chromophores were obtained from the Mulliken charges computed by the B3LYP/6‐31G(d) method. The point charges were placed and fixed at the center of the respective atoms. The geometrical parameters of the DIMER were obtained from the X‐ray structure of the special pair in the photosynthetic reaction center of *Rb. sphaeroides* at 2.6 Å resolution (PDB ID: 1AIG),^32^ as illustrated in Figure 2a. Hydrogen atoms were added with GaussView,^33^ and their positions were optimized at the B3LYP/cc‐pVDZ level of theory. To evaluate the parallel efficiencies of the QM unit of Platypus, the DIMER was computed with the HF method, DFT with B3LYP exchange–correlation functionals,^34^ and the (16e, 16o) CASCI^18^, ^19^ (CASCI(16,16)) method. The CASCI calculations were performed with the LOBPCG algorithm.^26^ The cc‐pVDZ basis sets^35^ were utilized for the HF and DFT calculations (2728 basis functions), and the 6‐31G** basis sets^36^ were used for the CASCI calculations (2728 basis functions). Both basis sets are often used in various studies. Although the numbers of basis functions of the cc‐pVDZ and 6‐31G** basis sets are same, the number of the primitive Gaussian functions of the cc‐pVDZ basis sets is larger than that of the 6‐31G** basis sets. The cc‐pVDZ basis sets are more appropriate for the measure of the speedup and parallelization ratio of the integral calculations and Fock matrices generation due to the larger computational costs. We performed the energy calculations with 1 to 65,536 core processors and the force calculations with 1 to 131,072 core processors at the HF and DFT levels of theory. In the energy calculations, we computed the Fock matrix with the direct SCF procedure twice by the HF method and once by the B3LYP method. The CASCI(16,16) calculations were also carried out with 2048 to 8192 core processors. In the CASCI(16, 16) calculations, the speedup and parallelization ratios were evaluated for the integral transformation from an AO basis to a MO basis and the diagonalization of the CI matrix by the direct CI method.^37^

For SIRIUS in water, the geometry of the chromophore was first optimized in the gas phase at the B3LYP/6‐31 + G(d) level, with the Gaussian09 program suites.^38^ The optimized structure was solvated in a 15 Å sphere of 449 TIP3P water molecules^39^ (1374 atoms) from the center of mass of the model (Fig. 2b). A harmonic potential of 100 kcal mol^−1^ Å^−2^ was applied to any water molecules that moved outside the sphere. The system was relaxed by using 10‐ns MD simulations with AMBER ff99 force fields,^21^ using the cosgene module of myPresto.^40^ We performed on‐the‐fly excited‐state QM/MM‐MD simulations on the system, using Platypus. The chromophore was chosen as the QM region in the simulations, while the surrounding water molecules were molecular‐mechanically treated, using the TIP3P water model.^39^ To compute the excited state of the chromophore, we employed the (8e, 8o) state‐averaged CASSCF, SA‐CASSCF(8,8), level of theory, in which one single set of molecular orbitals is used to compute all of the states of a given spatial and spin symmetry. The MINI‐4 basis sets^41^ were utilized to reduce the computational cost (87 basis functions). A time step of 0.25 fs was applied. The long‐range nonbonded interactions were truncated, using a 12 Å cutoff distance. The nonbonded interactions were updated at every step. We measured the computational time of 1000 steps of on‐the‐fly excited‐state QM/MM‐MD simulations at the SA‐CASSCF(8,8)/MINI‐4 level of theory, using 512, 1024, 2048, and 4096 core processors, and evaluated the speedups of the simulations. The SA‐CASSCF(8,8) calculations were performed with the LOBPCG algorithm.^26^ We performed the QM/MM‐MD simulation of 10,000 steps at 1 K for the relaxation of the excited state of the system. The system was then gradually heated from 1 to 300 K during 2.5 ps. Finally, we performed the 50‐ps QM/MM‐MD simulations (200,000 steps) at 300 K.

## Results and Discussion

### Speedup and parallelization ratio for energy calculations with the Platypus‐QM unit

The Platypus‐QM unit utilizes the eigen_sx routine of EigenExa^24^, ^25^ to solve the eigenvalue problems. We measured the parallel performance of EigenExa on the K computer, using 1 to 256 core processors, and compared it with the dsyevd subroutine of LAPACK.^42^ The size of the matrices to solve the eigenvalue problems was 2728 in the present study. The speedups and parallelization ratios are summarized in Table 1. The speedup kept increasing even when using 256 core processors. The parallelization ratio, however, provided the peak performance of 92.06% with 128 core processors. The dsyevd subroutine of LAPACK resulted in the elapsed time of 4.17 s, the speedup of 2.98, and the parallelization ratio of 75.95% using 8 core processors (Supporting Information Table S1), because LAPACK cannot use MPI to parallelize the subroutines. EigenExa using even 8 core processors was 1.2 times faster than LAPACK, and 2.89 times faster when 128 core processors were used. Here, we assigned 128 core processors to EigenExa, for the computation of the eigenvalue problems.

**Table 1:** Speedups and parallelization ratios for the computation of eigenvalue problems by EigenExa.

Number of CPUs	Number of threads	Number of cores	Elapsed time (sec)	Speedup	Parallelization ratio (%)
1	1	1	16.676	1.00	—
1	8	8	3.599	4.63	89.62
2	8	16	2.562	6.51	90.28
4	8	32	1.924	8.67	91.32
8	8	64	1.623	10.28	91.70
16	8	128	1.444	11.55	92.06
32	8	256	1.430	11.66	91.78

**Figure 3:**
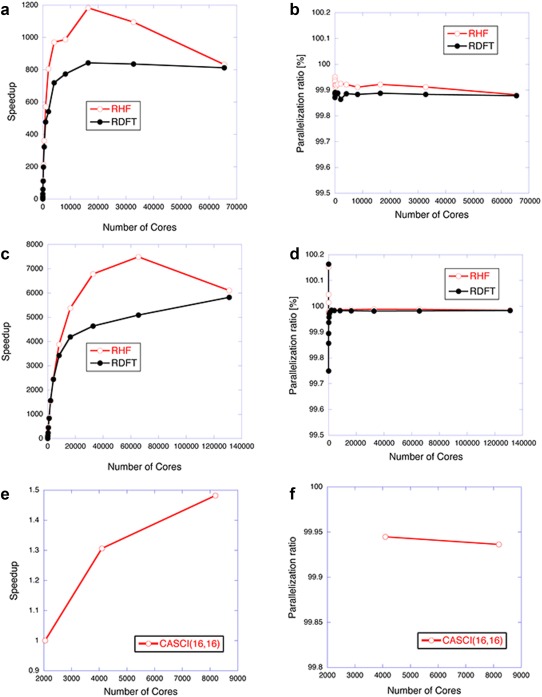
Speedups (**a**, **c**, and **e**) and parallelization ratios (**b**, **d**, and **f**) of energy (**a** and **b**) and force (**c** and **d**) calculations for the DIMER model at the HF/cc‐pVDZ and B3LYP/cc‐pVDZ levels of theory, and energy calculations for the DIMER at the CASCI(16,16)/6‐31G** (**e** and **f**) level of theory. In the CASCI(16,16) calculations, speedup and parallelization ratio were evaluated on the basis of the elapsed time measured with 2048 core processors. [Color figure can be viewed in the online issue, which is available at wileyonlinelibrary.com.]

The speedup and the parallelization ratio of the Platypus‐QM unit were evaluated to assess the parallel performance for the computation of the energies of DIMER, at the HF/cc‐pVDZ, B3LYP/cc‐pVDZ, and CASCI(16,16)/6‐31G** levels of theory. The evaluated speedups and parallelization ratios as functions of the core processors on the K computer are shown in Figure 3. The elapsed times, the speedups, and the ratios are also summarized in Supporting Information Tables S2 and S4. In the energy calculated by the HF method, since the subroutine of Fock matrices generation occupied about 95% of the computational time with a single core processor (19295.980 s), as shown in Supporting Information Table S2, the hybrid MPI and OpenMP parallel programming and the algorithm using SIMD of integrals enabled the Platypus‐QM unit to conduct the massively parallel computation with over 10,000 core processors. The Platypus‐QM unit provided the peak performance of the speedup and the parallelization ratio (1182.78% and 99.92%) with 16,384 core processors (2048 CPUs × 8 threads), as illustrated in Figures 3a and 3b. Since some subroutines for the orthogonalization of the MOs, the Cholesky decomposition, and the direct inversion in the iterative subspace (DIIS) are programmed with BLAS and LAPACK and are not parallelized with MPI in the Platypus‐QM unit, the MPI parallelization of these subroutines would further accelerate the Platypus‐QM unit. We also measured the thread parallel performance in a comparison between 64 (64 CPUs × 1 thread) and 512 (64 CPUs × 8 threads) core processors. The elapsed time for the computation was 373.72 s when using 64 core processors (64 CPUs × 1 thread), showing that the thread parallel performances of the speedup and the parallelization ratio by eight threads are 6.99% and 99.97%, respectively, and thus indicating that the speedup by using 64 CPUs is close to the ideal value (8.0) (Supporting Information Table S3).

As shown in Figures 3a and 3b and Supporting Information Table S4, the peak performance of the speedup and the parallelization ratio on the energy by the B3LYP/cc‐pVDZ method were evaluated to be 842.2% and 99.88% with 16,384 core processors (2048 CPUs × 8 threads). The thread parallel performance was also measured with 64 CPUs. The elapsed time for the computation was 247.25 s when using 64 core processors (64 CPUs × 1 thread), showing that the thread parallel performances of the speedup and the parallelization ratio by eight threads are 6.85% and 99.96%, respectively (Supporting Information Table S5). The maximum performances of the B3LYP method are smaller than those of the HF method, because the Fock matrix was computed twice in the HF calculations but only once in the B3LYP calculations.

The speedup and parallel ratios of the CASCI(16,16)/6‐31G** calculations were evaluated on the basis of the elapsed time with 2048 core processors, as shown in Figures 3e and 3f and Supporting Information Table S6. In the CASCI(16,16) calculation, since the size of the CI matrix was 34,764,300, remarkably large computational cost was required, and the integral evaluations, integral transformation, and the diagonalization of the CI matrix of the quantum calculation profit from large number of cores, despite of the relatively small number of atoms and basis functions. When 4 times larger number of core processors (8192 core processors (1024 CPUs × 8 threads)) was used, speedup is about 1.5, as shown in Figure 3e. The CASCI(16,16)/6‐31G** calculations showed that the speedup kept increasing up to 8192 core processors. Considering the difficulty in the parallelization of the CASCI calculations, these results can be judged to be fairly good. The CASCI(16,16) calculations showed that the parallelization ratio of 99.94% was larger than that obtained by the HF/cc‐pVDZ calculations, meaning that the CASCI computation has processes that can use the MPI parallelization (Figure 3f and Supporting Information Table S6). In the energy calculated by the CASCI method, the integral transformation (308.495 s) and solving the eigenvalue problem (289.848 s) were time‐consuming parts of the total computational time with a single core processor (691.328 s) rather than Fock matrices generation (5.516 s), as shown in Supporting Information Table S6. In the Platypus‐QM unit, integral transformation was highly parallelized, and the diagonalization of the CI matrix was accelerated by the direct CI method.^37^ We found superlinear speedup of integral transformation when using twice core processors (4096 core processors) than the reference (2048 core processors), because of the cache effect, as shown in Supporting Information Table S6.

### Speedup and parallelization ratio for force calculations with the Platypus‐QM unit

We also investigated the speedup and the parallelization ratio for the computation of the force with the Platypus‐QM unit. As shown in Figures 3c and 3d and Supporting Information Tables S7 and S9, the computational time of the two‐electron integral part of the force calculation accounted for about 98% of total elapsed time. The HF method provided the peak speedup of 7489.40 with 65,536 core processors (8192 CPUs × 8 threads), and the parallelization ratio was evaluated to be 99.988%. In the B3LYP calculations, the speedup and the parallelization ratio kept increasing even with 131,072 core processors (16,384 CPUs × 8 threads), as summarized in Figures 3c and 3d and Supporting Information Table S7 and S9, implying that the Platypus‐QM unit has been successfully designed to exhibit the high performance of massively parallel computers. These indicated that the force calculations of one‐ and two‐electron integral parts, which occupied almost all computational time (99.999%), were highly parallelized with the hybrid MPI/OpenMP parallelization. In particular, the two‐electron integral parts showed superlinear speedup, as shown in Supporting Information Tables S7 and S9. In the thread parallel performance for the computation of the forces of the DIMER, the speedups and the parallelization ratios were almost ideal (7.96 and 7.90 for speedups and 99.999% and 99.997% for parallelization ratios by the HF and B3LYP calculations, respectively) (Supporting Information Tables S8 and S10).

### On‐the‐fly excited‐state QM/MM‐MD simulations of SIRIUS in water

There are several QM/MM programs, but as far as we know, there is no study where a QM/MM‐MD simulation is performed on the fly to compute the excited state. We evaluated the parallel performance of on‐the‐fly excited‐state QM/MM‐MD simulations of the SIRIUS system, at the SA‐CASSCF(8,8) level of theory. The elapsed time for the simulation was measured with 512, 1024, 2048, and 4096 core processors of the K computer. As summarized in Table 2, the calculation with 4096 core processors was 1.6 times faster than that with 512 core processors, and the speedup obviously kept increasing even with 4096 core processors, indicating that Platypus can be expected to perform the excited‐state QM/MM‐MD simulations for fluorescent proteins.

**Table 2:** Speedups for 1000 step on‐the‐fly excited‐state QM/MM‐MD simulations at the SA‐CASSCF(8,8)/MINI‐4 level of theory.

Number of CPUs	Number of threads	Number of cores	Elapsed time (sec)	Speedup
64	8	512	30155.600	1
128	8	1024	22864.020	1.32
256	8	2048	19900.071	1.52
512	8	4096	18909.481	1.60

**Figure 4:**
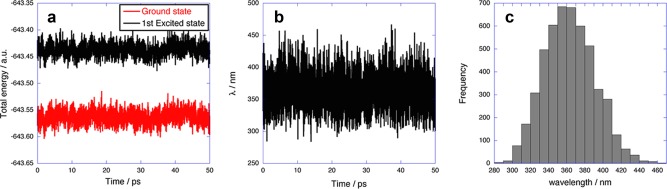
Ground‐state and excited state energies (**a**) and emission energies (**b**) of the SIRIUS system in the 50‐ps on‐the‐fly QM/MM‐MD simulation at the SA‐CASSCF(8,8)/MINI‐4 level of theory, as a function of time. Histogram of the emission energies (**c**) of the SIRIUS system in the 50‐ps on‐the‐fly QM/MM‐MD simulation at the SA‐CASSCF(8,8)/MINI‐4 level of theory. The bin width is 10 nm. [Color figure can be viewed in the online issue, which is available at wileyonlinelibrary.com.]

We conducted 50‐ps on‐the‐fly excited‐state QM/MM‐MD simulations of the system, at the SA‐CASSCF(8,8) level of theory. Figures 4a and 4b show the total energies in the ground and excited states and the emission energies of SIRIUS in the QM/MM‐MD simulation, respectively. As shown in Figure 4a, the standard deviations of the total energies were 0.32 and 0.31 eV in the ground and excited states, respectively, indicating that Platypus produced a stable on‐the‐fly excited‐state QM/MM‐MD simulation during 50 ps. The emission energies computed in the 50 ps simulation showed the Gaussian distribution with the mean emission energy of 363 nm and the standard deviation of 28 nm (3.43 ± 0.26 eV), as illustrated in Figure 4c. The minimum emission energy was 2.66 eV (466 nm). As compared to the experimental emission peak,^31^ although our computation underestimated the emission wavelength by 100 nm, because the poor basis sets destabilized the excited state, the longest emission wavelength (466 nm) was close to the experimental value.

## Concluding Remarks

A massively parallel program of quantum mechanical‐molecular mechanical (QM/MM) molecular dynamics simulations, Platypus (PLATform for dYnamic Protein Unified Simulation), has been developed for the elucidation of protein functions. The performances of Platypus were assessed for DIMER and SIRIUS in water on the K computer. The Platypus‐QM unit exhibited the increase in speedup up to 20,000 core processors at the HF/cc‐pVDZ, B3LYP/cc‐pVDZ levels of theory, and up to 10,000 core processors at the CASCI(16,16)/6‐31G** level of theory, on the K computer. Platypus accelerated on‐the‐fly excited‐state QM/MM‐MD simulations for SIRIUS in water, using over 4000 core processors.

## Supporting information

Supporting Information
