# Supplementary material for: Development of massive multilevel molecular dynamics simulation program, platypus (PLATform for dYnamic protein unified simulation), for the elucidation of protein functions
Source: J Comput Chem. 2016 Mar 4;37(12):1125–32. doi: 10.1002/jcc.24318 (PMC4825406; doi:10.1002/jcc.24318)
Supplement: Supplementary file 1 — Supporting Information [file JCC-37-1125-s001.pdf]

Supplementary Materials

Development of massive multi-level molecular dynamics simulation program,

Platypus (PLATform for dYnamic Protein Unified Simulation),

for the elucidation of protein functions

Yu Takano,* Kazuto Nakata, Yasushige Yonezawa, and Haruki Nakamura

Table S1. Speedups and parallelization ratios for the computation of eigenvalue problems by LAPACK (dsyevd).

Number of CPUs	Number of threads	Number of cores	Elapsed time [sec]	Speedup[a]	Serial fraction [%][b]	Parallelization ratio [%][c]
1	1	1	12.448	1.000	—	—
1	8	8	4.175	2.981	25.047	75.953

[a] Speedup was evaluated by using eq. 3. Here, the elapsed time measured with a single core processor was used as T_1.

[b] Serial fraction was evaluated by using eq. 5.

[c] Parallelization ratio was evaluated by using eq. 4. Here, the elapsed time measured with a single core processor was used as T_1.

Table S2. Speedups and parallelization ratios for energy calculations of DIMER at the HF/cc-pVDZ level of theory.

Number of CPUs	Number of threads	Number of cores	Elapsed time[sec]		Speedup [a]		Serial fraction[%] [b]		Parallelization ratio[%] [c]	
			Total	Fock matrix generation	Total	Fock matrix generation	Total	Fock matrix generation	Total	Fock matrix generation
1	1	1	19295.980	18261.522	1.000	1.000	—	—	—	—
1	8	8	2420.357	2283.530	7.972	7.997	0.0495	0.0053	99.9505	99.9947
2	8	16	1217.018	1142.758	15.855	15.980	0.0609	0.0083	99.9391	99.9917
4	8	32	615.492	572.010	31.351	31.925	0.0668	0.0076	99.9332	99.9924
8	8	64	316.704	285.195	60.927	64.032	0.0800	-0.0008	99.9200	100.0008
16	8	128	167.661	144.157	115.089	126.678	0.0883	0.0082	99.9117	99.9918
32	8	256	90.798	73.735	212.517	247.663	0.0802	0.0132	99.9198	99.9868
64	8	512	53.444	37.804	361.053	483.052	0.0818	0.0117	99.9182	99.9883
128	8	1024	33.719	19.239	572.265	949.170	0.0772	0.0077	99.9228	99.9923
256	8	2048	23.971	10.297	804.971	1773.527	0.0754	0.0076	99.9246	99.9924
512	8	4096	19.893	5.836	969.964	3129.348	0.0787	0.0075	99.9213	99.9925
1024	8	8192	19.538	4.063	987.629	4495.108	0.0891	0.0100	99.9109	99.9900
2048	8	16384	16.314	2.922	1182.783	6249.603	0.0784	0.0099	99.9216	99.9901

[a] Speedup was evaluated by using eq. 3. Here, the elapsed time measured with a single core processor was used as T_1.

[b] Serial fraction was evaluated by using eq. 5.

[c] Parallelization ratio was evaluated by using eq. 4. Here, the elapsed time measured with a single core processor was used as T_1.

Table S3. Comparison of speedups and parallelization ratios for energy calculations of DIMER between 64 (64 CPUs x 1 thread) and 512 (64 CPUs x 8 threads) core processors at the HF/cc-pVDZ level of theory.

			Elapsed time[sec]		Speedup[a,b]		Serial fraction[%][a,c]		Parallelization ratio[%][a,d]	
Number of CPUs	Number of threads	Number of cores	Total	Fock matrix generation	Total	Fock matrix generation	Total	Fock matrix generation	Total	Fock matrix generation
64	1	64	373.727	300.942	1.000	1.000	—	—	—	—
64	8	512	53.444	37.804	6.993	7.960	0.0328	0.0011	99.9672	99.9989

[a] Speedup, serial fraction, and parallelization ratio were evaluated on the basis of the elapsed time measured with 64 core processors.

[b] Speedup was evaluated by using eq. 3. Here, the elapsed time measured with 64 core processors was used as T_1.

[c] Serial fraction was evaluated by using eq. 5.

[d] Parallelization ratio was evaluated by using eq. 4. Here, the elapsed time measured with 64 core processors was used as T_1.

Table S4. Speedups and parallelization ratios for energy calculations of DIMER at the B3LYP/cc-pVDZ level of theory.

Number of CPUs	Number of threads	Number of cores	Elapsed time[sec] Total	Elapsed time[sec] Fock matrix generation	Speedup[a] Total	Speedup[a] Fock matrix generation	Serial fraction[%][b] Total	Serial fraction[%][b] Fock matrix generation	Parallelization ratio[%][c] Total	Parallelization ratio[%][c] Fock matrix generation
1	1	1	11657.211	10618.977	1.000	1.000	—	—	—	—
1	8	8	1470.312	1335.344	7.928	7.952	0.1290	0.0858	99.8710	99.9142
2	8	16	740.755	667.288	15.737	15.914	0.1114	0.0362	99.8886	99.9638
4	8	32	377.119	334.818	30.911	31.716	0.1136	0.0289	99.8864	99.9711
8	8	64	194.683	166.871	59.878	63.636	0.1093	0.0091	99.8907	99.9909
16	8	128	105.012	84.276	111.009	126.002	0.1205	0.0125	99.8795	99.9875
32	8	256	59.014	43.065	197.532	246.582	0.1161	0.0150	99.8839	99.9850
64	8	512	36.094	22.083	322.972	480.860	0.1145	0.0127	99.8855	99.9873
128	8	1024	24.425	11.271	477.267	942.127	0.1120	0.0085	99.8880	99.9915
256	8	2048	21.548	6.400	540.980	1659.103	0.1361	0.0115	99.8639	99.9885
512	8	4096	16.207	3.532	719.258	3006.898	0.1146	0.0088	99.8854	99.9912
1024	8	8192	15.062	2.312	773.948	4592.288	0.1170	0.0096	99.8830	99.9904
2048	8	16384	13.841	1.798	842.204	5905.298	0.1126	0.0108	99.8874	99.9892

[a] Speedup was evaluated by using eq. 3. Here, the elapsed time measured with a single core processor was used as T_1.

[b] Serial fraction was evaluated by using eq. 5.

[c] Parallelization ratio was evaluated by using eq. 4. Here, the elapsed time measured with a single core processor was used as T_1.

Table S5. Comparison of speedups and parallelization ratios for energy calculations of DIMER between 64 (64 CPUs x 1 thread) and 512 (64 CPUs x 8 threads) core processors at the B3LYP/cc-pVDZ level of theory.

			Elapsed time[sec]		Speedup[a,b]		Serial fraction[%][a,c]		Parallelization ratio[%][a,d]	
Number of CPUs	Number of threads	Number of cores	Total	Fock matrix generation	Total	Fock matrix generation	Total	Fock matrix generation	Total	Fock matrix generation
64	1	64	247.253	176.085	1.000	1.000	—	—	—	—
64	8	512	36.094	22.083	6.850	7.974	0.0384	0.0007	99.9616	99.9993

[a] Speedup, serial fraction, and parallelization ratio were evaluated on the basis of the elapsed time measured with 64 core processors.

[b] Speedup was evaluated by using eq. 3. Here, the elapsed time measured with 64 core processors was used as T_1.

[c] Serial fraction was evaluated by using eq. 5.

[d] Parallelization ratio was evaluated by using eq. 4. Here, the elapsed time measured with 64 core processors was used as T_1.

Table S6. Speedups and parallelization ratios for energy calculations of DIMER at the CASCI(16,16)/6-31G** level of theory.

Number of CPUs	Number of threads	Number of cores	Elapsed time[sec] Total	Elapsed time[sec] Integral transformation	Elapsed time[sec] Fock matrix generation	Elapsed time[sec] Eigenvalue problem	Speedup[a,b] Total	Speedup[a,b] Integral transformation	Speedup[a,b] Fock matrix generation	Speedup[a,b] Eigenvalue problem
256	8	2048	691.328	308.495	5.516	289.848	1.000	1.000	1.000	1.000
512	8	4096	529.307	145.659	3.081	171.417	1.306	2.118	1.790	1.691
1024	8	8192	466.399	105.787	2.033	142.372	1.482	2.916	2.713	2.036

Serial fraction[%][a,c] Total	Serial fraction[%][a,c] Integral transformation	Serial fraction[%][a,c] Fock matrix generation	Serial fraction[%][a,c] Eigenvalue problem	Parallelization ratio[%][a] Total	Parallelization ratio[%][a] Integral transformation	Parallelization ratio[%][a] Fock matrix generation	Parallelization ratio[%][a] Eigenvalue problem
—	—	—	—	—	—	—	—
0.0553	-0.0026	0.0065	0.0109	99.9447	100.0026	99.9935	99.9891
0.0637	0.0069	0.0092	0.0231	99.9363	99.9931	99.9908	99.9769

[a] Speedup, serial fraction, and parallelization ratio were evaluated on the basis of the elapsed time measured with 2048 core processors.

[b] Speedup was evaluated by using eq. 3. Here, the elapsed time measured with 2048 core processors was used as T_1.

[c] Serial fraction was evaluated by using eq. 5.

[d] Parallelization ratio was evaluated by using eq. 4. Here, the elapsed time measured with 2048 core processors was used as T_1.

Table S7. Speedups and parallelization ratios for force calculations of DIMER at the HF/cc-pVDZ level of theory.

			Elapsed time[sec]			Speedup[a]			Serial fraction[%][b]			Parallelization ratio[%][c]		
Number of CPUs	Number of threads	Number of cores	Total	1-electron integral	2-electron integral	Total	1-electron integral	2-electron integral	Total	1-electron integral	2-electron integral	Total	1-electron integral	2-electron integral
1	1	1	55866.363	1366.995	54498.802	1.000	1.000	1.000	—	—	—	—	—	—
1	8	8	6909.150	172.495	6735.949	8.086	7.925	8.091	-0.1517	0.1355	-0.1602	100.1517	99.8645	100.1602
2	8	16	3467.883	86.162	3381.134	16.110	15.865	16.118	-0.0454	0.0565	-0.0490	100.0454	99.9435	100.0490
4	8	32	1738.001	43.394	1694.092	32.144	31.502	32.170	-0.0145	0.0510	-0.0170	100.0145	99.9490	100.0170
8	8	64	885.490	22.065	862.910	63.091	61.954	63.157	0.0229	0.0524	0.0212	99.9771	99.9476	99.9788
16	8	128	449.616	11.380	437.715	124.254	120.127	124.508	0.0237	0.0516	0.0221	99.9763	99.9484	99.9779
32	8	256	229.790	6.129	223.142	243.119	223.031	244.234	0.0208	0.0580	0.0189	99.9792	99.9420	99.9811
64	8	512	119.173	3.489	115.157	468.782	391.846	473.255	0.0180	0.0600	0.0160	99.9820	99.9400	99.9840
128	8	1024	64.038	2.153	61.368	872.400	635.000	888.071	0.0170	0.0599	0.0150	99.9830	99.9401	99.9850
256	8	2048	36.330	1.953	33.425	1537.740	699.868	1630.466	0.0162	0.0941	0.0125	99.9838	99.9059	99.9875
512	8	4096	22.742	1.347	20.105	2456.563	1014.558	2710.729	0.0163	0.0742	0.0125	99.9837	99.9258	99.9875
1024	8	8192	14.401	1.132	12.236	3879.423	1207.309	4454.078	0.0136	0.0706	0.0102	99.9864	99.9294	99.9898
2048	8	16384	10.394	1.120	8.455	5375.072	1220.981	6446.052	0.0125	0.0758	0.0094	99.9875	99.9242	99.9906
4096	8	32768	8.231	0.945	5.830	6787.285	1447.136	9347.254	0.0117	0.0661	0.0076	99.9883	99.9339	99.9924
8192	8	65536	7.459	1.086	5.235	7489.404	1258.595	10411.361	0.0118	0.0779	0.0081	99.9882	99.9221	99.9919
16384	8	131072	9.153	0.985	4.406	6103.336	1387.374	12368.753	0.0156	0.0713	0.0073	99.9844	99.9287	99.9927

[a] Speedup was evaluated by using eq. 3. Here, the elapsed time measured with a single core processor was used as T_1.

[b] Serial fraction was evaluated by using eq. 5.

[c] Parallelization ratio was evaluated by using eq. 4. Here, the elapsed time measured with a single core processor was used as T_1.

Table S8. Comparison of speedup and parallelization ratios for force calculations of DIMER between 64 (64 CPUs x 1 thread) and 512 (64 CPUs x 8 threads) core processors at the HF/cc-pVDZ level of theory.

Number of CPUs	Number of threads	Number of cores	Elapsed time[sec]			Speedup[a,b]		
			Total	1-electron integral	2-electron integral	Total	1-electron integral	2-electron integral
64	1	64	948.643	23.281	924.846	1.000	1.000	1.000
64	8	512	119.173	3.489	115.157	7.960	6.673	8.031

Serial fraction[%][a,c]			Parallelization ratio[%][a,d]		
Total	1-electron integral	2-electron integral	Total	1-electron integral	2-electron integral
—	—	—	—	—	—
0.0011	0.0456	-0.0009	99.9989	99.9544	100.0009

[a] Speedup, serial fraction, and parallelization ratio were evaluated on the basis of the elapsed time measured with 64 core processors.

[b] Speedup was evaluated by using eq. 3. Here, the elapsed time measured with 64 core processors was used as T_1.

[c] Serial fraction was evaluated by using eq. 5.

[d] Parallelization ratio was evaluated by using eq. 4. Here, the elapsed time measured with 64 core processors was used as T_1.

Table S9. Speedup and parallelization ratios of force calculations of DIMER at the B3LYP/cc-pVDZ level of theory.

Number of CPUs	Number of threads	Number of cores	Elapsed time[sec]			Speedup[a]			Serial fraction[%][b]			Parallelization ratio[%][c]		
			Total	1-electron integral	2-electron integral	Total	1-electron integral	2-electron integral	Total	1-electron integral	2-electron integral	Total	1-electron integral	2-electron integral
1	1	1	57456.397	1367.819	56087.529	1.000	1.000	1.000	—	—	—	—	—	—
1	8	8	7099.900	171.568	6927.539	8.093	7.972	8.096	-0.1634	0.0494	-0.1699	100.1634	99.9506	100.1699
2	8	16	3726.127	86.030	3485.639	15.420	15.899	16.091	0.2508	0.0422	-0.0377	99.7492	99.9578	100.0377
4	8	32	1875.301	43.286	1744.087	30.638	31.599	32.159	0.1433	0.0409	-0.0159	99.8567	99.9591	100.0159
8	8	64	957.335	21.943	863.837	60.017	62.336	64.928	0.1053	0.0424	-0.0227	99.8947	99.9576	100.0227
16	8	128	484.818	11.262	441.570	118.511	121.458	127.018	0.0630	0.0424	0.0061	99.9370	99.9576	99.9939
32	8	256	249.014	6.005	219.130	230.736	227.768	255.955	0.0429	0.0486	0.0001	99.9571	99.9514	99.9999
64	8	512	129.212	3.847	112.522	444.666	355.597	498.457	0.0296	0.0861	0.0053	99.9704	99.9139	99.9947
128	8	1024	68.926	2.409	57.419	833.594	567.851	976.816	0.0223	0.0785	0.0047	99.9777	99.9215	99.9953
256	8	2048	36.738	1.352	28.602	1563.951	1011.786	1960.982	0.0151	0.0500	0.0022	99.9849	99.9500	99.9978
512	8	4096	23.546	1.096	18.859	2440.187	1247.992	2974.001	0.0166	0.0557	0.0092	99.9834	99.9443	99.9908
1024	8	8192	16.768	1.223	13.876	3426.456	1118.870	4042.147	0.0170	0.0772	0.0125	99.9830	99.9228	99.9875
2048	8	16384	13.715	1.153	10.789	4189.263	1185.916	5198.529	0.0178	0.0782	0.0131	99.9822	99.9218	99.9869
4096	8	32768	12.394	1.132	9.348	4635.970	1208.062	5999.909	0.0185	0.0797	0.0136	99.9815	99.9203	99.9864
8192	8	65536	11.291	1.306	8.204	5088.483	1047.126	6836.358	0.0181	0.0940	0.0131	99.9819	99.9060	99.9869
16384	8	131072	9.873	0.798	7.508	5819.641	1713.486	7470.565	0.0164	0.0576	0.0126	99.9836	99.9424	99.9874

[a] Speedup was evaluated by using eq. 3. Here, the elapsed time measured with a single core processor was used as T_1.

[b] Serial fraction was evaluated by using eq. 5.

[c] Parallelization ratio was evaluated by using eq. 4. Here, the elapsed time measured with a single core processor was used as T_1.

Table S10. Comparison of speedups and parallelization ratios for force calculations of DIMER between 64 (64 CPUs x 1 thread) and 512 (64 CPUs x 8 threads) core processors at the B3LYP/cc-pVDZ level of theory.

Number of CPUs	Number of threads	Number of cores	Elapsed time[sec]			Speedup[a,b]		
			Total	1-electron integral	2-electron integral	Total	1-electron integral	2-electron integral
64	1	64	1020.217	23.280	900.466	1.000	1.000	1.000
64	8	512	129.212	3.847	112.522	7.896	6.052	8.003

Serial fraction[%][a,c]			Parallelization ratio[%][a,d]		
Total	1-electron integral	2-electron integral	Total	1-electron integral	2-electron integral
—	—	—	—	—	—
0.0030	0.0752	-0.0001	99.9970	99.9248	100.0001

[a] Speedup, serial fraction, and parallelization ratio were evaluated on the basis of the elapsed time measured with 64 core processors.

[b] Speedup was evaluated by using eq. 3. Here, the elapsed time measured with 64 core processors was used as T_1.

[c] Serial fraction was evaluated by using eq. 5.

[d] Parallelization ratio was evaluated by using eq. 4. Here, the elapsed time measured with 64 core processors was used as T_1.
